# Serum Splicing Factor Proline- and Glutamine-Rich Is a Diagnostic Marker for Non-Small-Cell Lung Cancer and Other Solid Cancers

**DOI:** 10.3390/ijms25168766

**Published:** 2024-08-12

**Authors:** Libang Yang, Adam Gilbertsen, Blake Jacobson, Robert Kratzke, Craig A. Henke

**Affiliations:** 1 Department of Medicine, University of Minnesota, Minneapolis, MN 55455, USA; gilbe398@umn.edu (A.G.);; 2 Hematology, Oncology and Transplantation, School of Medicine, University of Minnesota, 420 Delaware Street, SE, Minneapolis, MN 55455, USA; jacob023@umn.edu (B.J.); kratz003@umn.edu (R.K.)

**Keywords:** NSCLC, SFPQ, diagnostic marker, solid cancers

## Abstract

Cancer markers are measurable molecules in blood or tissues that are produced by tumor cells or immune cells in response to cancer progression. They play an important role in clinical diagnosis, prognosis, and therapy monitoring. Splicing factor proline- and glutamine-rich (SFPQ) plays an important role in cancer growth and metastasis. SFPQ is not only more highly expressed in non-small-cell lung cancer (NSCLC) cells than it is in controls, but also highly expressed in cancer cells in patients with other solid cancers. Thus, a new enzyme-linked immunosorbent assay (ELISA) for detecting SFPQ was developed, in which the SFPQ protein is trapped by the first specific mAb coated on a microplate, and then recognized by a second specific mAb. This assay allows for the specific detection of SFPQ in the serum of patients with solid cancer. Regarding NSCLC, the serum SFPQ levels distinguished the non-cancer controls from the patients with NSCLC, with an area under the curve of 0.876, a sensitivity of 87%, and a specificity of 94%. The serum SFPQ levels were significantly elevated in the patients with NSCLC or other solid cancers. In conclusion, serum SFPQ could be a promising novel diagnostic biomarker for NSCLC and other malignancies.

## 1. Introduction

Cancer markers are measurable molecules that circulate in blood or tissues, are expressed by tumor cells or immune cells, and are involved in cancer progression. Cancer markers are crucial for clinical diagnosis, prognosis, and cancer therapy monitoring. The early detection of cancer and the monitoring of cancer progression are crucial for reducing cancer mortality worldwide [1,2,3,4]. In recent years, various biomolecules and molecular complexes, including exosomes, circulating tumor DNA, and serum noncoding RNAs, have been identified as diagnostic biomarkers; however, these methods are generally technically complex and expensive, which hinders their utility in clinical applications. Thus, there is still a need to identify accurate serum diagnostic biomarkers that can be routinely and inexpensively measured for the large-scale screening of early-stage cancer and monitoring cancer progression, particularly solid cancers. Serological tumor biomarkers are desirable for cancer screening because of the easy and relatively noninvasive method of sample collection and the low cost [4,5]. However, the conventional serum tumor markers, such as PSA, carcinoembryonic antigen (CEA), and Her2, are not satisfactory due to their relatively low sensitivity and specificity. Non-small-cell lung cancer (NSCLC) is one of the most common cancers that often leads to death. Several proteins could potentially serve as diagnostic markers for lung cancer [6,7,8,9].

SFPQ is a multifunctional protein. It has the ability to bind to DNA and RNA, and thus regulate RNA splicing and protein transcription [10,11,12]. It is highly expressed in many cancers and plays critical roles in those cancers. It has also been discovered that its accumulation in the cytoplasm could be the cause of some neuronal diseases [13,14,15,16]. Our previous work demonstrated the SFPQ level was much higher in NSCLC samples with a limited sample size [17]. Here, we further observed high-level SFPQ expression in the cancer cells and tissues from patients with NSCLC or other solid cancers. In addition, we developed a sandwich ELISA to detect SFPQ in cell lysates, cell media, and human sera. Employing this novel ELISA, we measured SFPQ and evaluated the diagnostic significance of the serum SFPQ levels in patients with cancer, with a particular focus on solid cancers.

## 2. Results

### 2.1. Identification of SFPQ as a Candidate Diagnostic Biomarker in Solid Cancers

**Figure 1:**
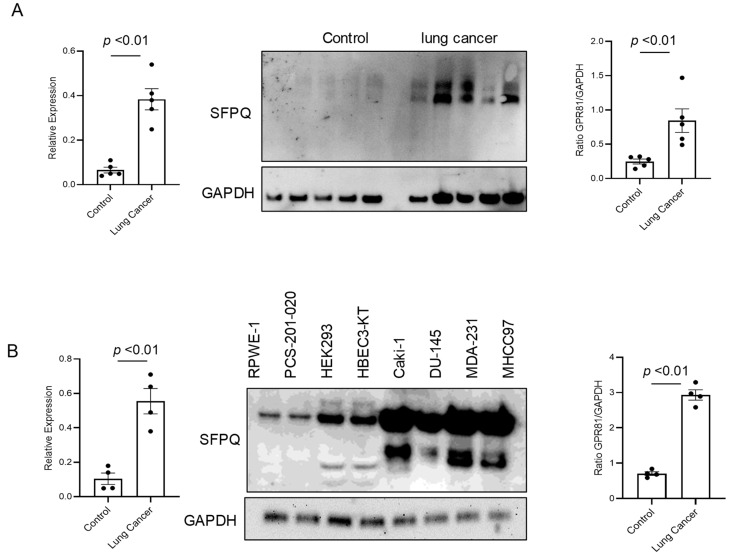
The levels of SFPQ are different between the patients with NSCLC, the patients with other solid cancers, and the controls. The SFPQ levels were analyzed by RT-PCR (left panel) and Western blot analysis (middle). The densitometry values are shown in the right hand graph. The multiple SFPQ bands present in NSCLC (**A**), other solid cancers (**B**), and the controls are the different SFPQ isoforms. The Western blot analysis of the primary cell lysates revealed that the SFPQ expression levels differed between the control and NSCLC groups. GAPDH was used as a loading marker.

**Figure 2:**
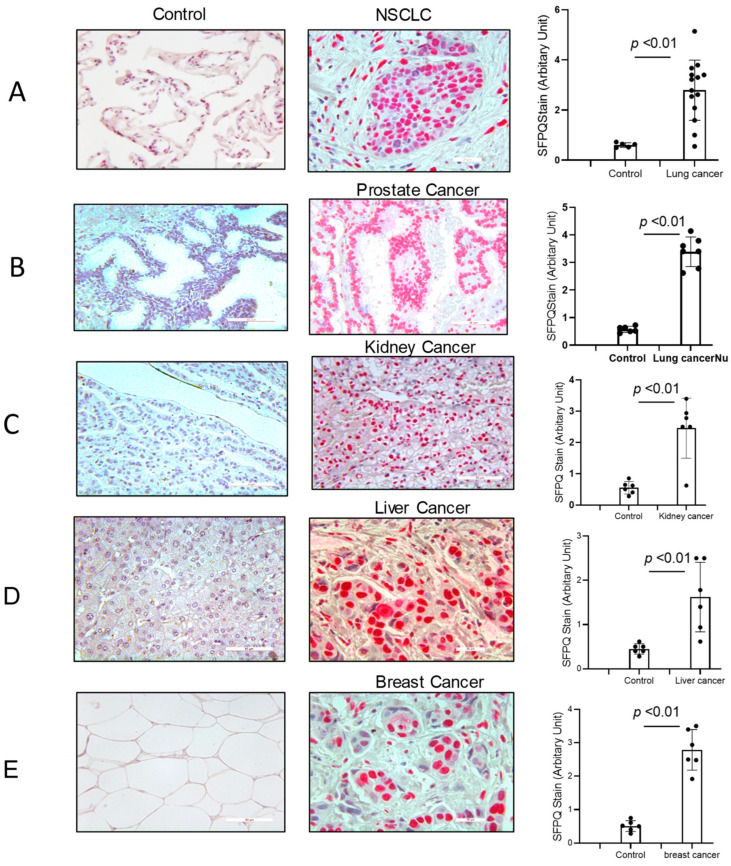
SFPQ exists in the cancer cells from NSCLC and the other solid cancers. Immunohistochemistry (IHC) staining was performed on the human NSCLC lung tissue, the other solid cancer tissue, and the control tissues. SFPQ antibodies were used to detect SFPQ in the samples. HPA054689 (polyclonal antigen from Invitrogen) was used on the control and cancer tissue sections (red); the counter stain was Mayer’s hematoxylin, which used to display nuclei (blue). Scale bar = 100 µm. (**A**). The IHC staining of lung cancer and control tissues was performed. These example images show the control tissue (left panel) and the lung cancer tissue (middle). Semi-quantification is summed in the right figure, performed with Image J (v1.8.0). (**B**). Prostate cancer. (**C**). Kidney cancer. (**D**). Liver cancer. (**E**). Breast cancer.

**Figure 3:**
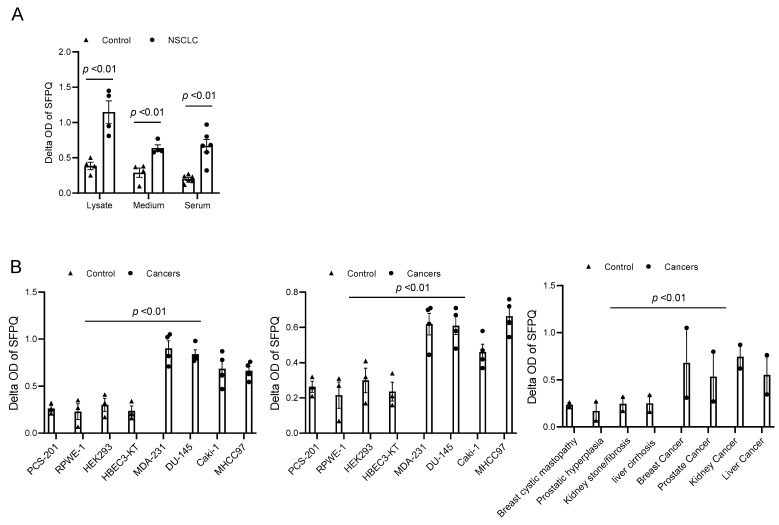
SFPQ levels are high in NSCLC and other cancer cell lysates and cell culture conditioned media. (**A**). SFPQ ELISA can detect SFPQ in samples of cell lysate, cell culture conditioned medium from cancer, and serum from controls and patients with NSCLC. N = 4; controls, PCS-201; 203, HLF 271, HLF 279; NSCLC cells (A549, H661, H858, and H645). Serum from 6 random controls and patients with NSCLC was taken randomly. (**B**). Measurements of SFPQ in control and cancer cell lysates (left panel), cell culture conditioned medium (middle panel) from cancer, and serum from controls and patients with other solid cancers were taken via ELISA. N = 4; controls, PCS-201; RPWE-1, HEK293, and HBEC3-KT; cancer cells (Caki-1, DU-145, MDA231, and MHCC97). Right panel: serum from 8 controls and 2 patients with liver, kidney, prostate, and breast cancers was taken randomly.

SFPQ has been reported to be highly expressed and to promote cell growth in neuronal disease and NSCLC, and we further detected the SFPQ levels in other solid cancer cells. We found that SFPQ is highly expressed in NSCLC or the other solid cancer cells. SFPQ is highly expressed not only in NSCLC cells (Figure 1A), but also in prostate Du145 cells, breast cancer MDA231 cells, kidney cancer Caki-1 cells, and liver cancer MHCC97 cells, according to both RT-PCR and Western blot analysis (Figure 1B). IHC was conducted on the tissue sections from patients with solid cancer. When we quantified the SFPQ staining area and density in the control and solid cancer tissue sections, the amount of NSCLC cancer staining was five times greater than that in the control tissue (Figure 2). The level of the SFPQ stain was also much greater in the cancer cells from the other solid cancer tissue than that in the control cells. To observe if SFPQ could be released out the cells, a cell culture conditioned medium was used to detect SFPQ using ELISA. The SFPQ levels measured by ELISA were greater in the cancer lysates and culture media than those in the controls. We further measured SFPQ in the human serum from the patients with NSCLC and other diseases. The SFPQ serum levels were higher in the cancer serum than in the serum from the patients with the other diseases (Figure 3A). The SFPQ levels were higher in the cell lysate and cell culture conditioned medium from the prostate Du145 cells, the breast cancer MDA231 cells, the kidney cancer Caki-1 cells, and the liver cancer MHCC97 cells than those from the control cells. The SFPQ serum levels in prostate cancer, breast cancer, kidney cancer and liver cancer are higher than those in the control serum (Figure 3B). Thus, the SFPQ level is not only high in cancer cells, but it also is released out of the cancer cells. It could serve as a clinical diagnostic marker.

### 2.2. Monoclonal Antibodies Specific for SFPQ

SFPQ is highly expressed in cancer and can be detected in cell culture media, which suggests that SFPQ can be released into the body fluids of patients with cancer. We started to generate monoclonal antibodies (mAbs) using purified human SFPQ. In order to capture all the SFPQ isoforms, antibodies against SFPQ, P23246 (Raybiotech, Peachtree Corners, GA, USA), HPA054689 (Sigma, St. Louis, MO, USA), and 67129-1-Ig (Proteintech, Rosemont, IL, USA), were used to make affinity columns to purify SFPQ from the cancer cell lines A549, Du145, H661, and PCS-201. The purified SFPQ was then used to immunize mice and prepare the hybridomas [18,19,20]. The hybridomas that were found to be positive upon screening on the SFPQ-coated plates were further tested for recombinant (Abnova, Taipei, Taiwan) and purified human SFPQ. Among them, only eight (1A6, 2A4, 2C1, 2D5, 2F8, 3A7, 3D5, and 4A1) recognized both the recombinant human and purified human SFPQ.

### 2.3. SFPQ/mAbs Evaluation for ELISA Development

**Figure 4:**
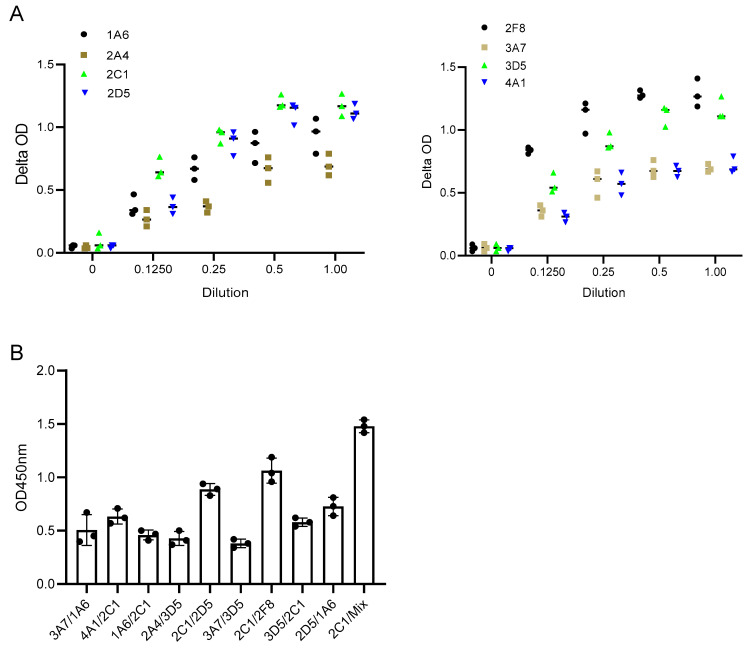
Characterization of Anti-SFPQ mAbs. (**A**). Representative dynamic binding of Anti-SFPQ mAbs. Coating: 2 ug/mL purified native SFPQ and 50 ng/mL mAb, biotinylated. (**B**). Test of different mAbs combinations for ELISA. Different combinations of coated/biotinylated mAbs were tested for ELISA using 2C1, 2D5, or 2F8 antibodies and recombinant SFPQ in crude culture medium of insect cells. Streptavidin–HRP and TMB chromogenic substrates were then added to well, and optic density (OD) was read at 450 nm after stopping enzymatic reaction with H_2_SO_4_. This test was conducted twice with identical results. We subsequently performed ELISA experiments with either one of two combinations: coated 2D5 or 2F8 with biotinylated 2C1. We also performed assays with coated 2D5 + 2F8 mix combined with biotinylated 2C1 for optimized detection of SFPQ (see below).

In this study, the binding pattern of Anti-SFPQ mAbs was evaluated using a pairwise binding approach, in which the ability of the pairs of antibodies to bind to the SFPQ antigen was tested. Antibodies that are directed toward distinct epitopes can be used for the development of assays, while those that are directed toward the same epitopes or close epitopes cannot. Additionally, antibodies with high affinity are another factor to consider for assay development. One was used as the coating antibody, and the other was used as the detecting antibody, which was labeled with biotin before use. The biotinylation of the antibody was performed using the EZ-Link™Sulfo-NHS-LC Biotinylation Kit (21435, Thermo Fisher Scientific, Waltham, MA, USA) following the manufacturer’s instructions. Some of the combinations had better signals than the others (Figure 4). 2C1/2F8 or 2D5 was selected for the development of the assay.

Optimization and development of dual mAb-based sandwich ELISA. To improve signal amplification, the ELISA protocol was optimized using the biotin–streptavidin conjugation strategy. In fact, the use of the high-affinity and noncovalent interaction between biotin and streptavidin produced a maximized signal-to-noise ratio with increased sensitivity compared to that of the conventional enzyme-labeled secondary antibodies.

To exploit the biotin–streptavidin conjugation strategy, the SFPQ antibodies labeled with biotin (IgG:Biotin =1:6 ratio) were evaluated. The antibodies with relative high affinity were identified and selected for further assay development in order to develop a highly sensitive assay. An assay using 2F8 as the capture antibody (3 μg/mL) and biotinylated 2C1 as the detection agent (0.1 μg/mL) was identified as the best configuration in terms of the curve range (0.19–12.10 ng/mL) and the coefficient of determination (R2) (0.995).

Theoretically, the ability of polyclonal antibodies to bind multiple epitopes allows for signal amplification in samples with low levels of expression of the target protein. Unfortunately, no polyclonal antibodies have been developed using native human SFPQ. We compared two antibody combinations to a single antibody in the assay, and there was a better signal in the assay with the antibody combination (Figure 4). This finding suggested that an assay with polyclonal antibodies may provide a better signal.

**Figure 5:**
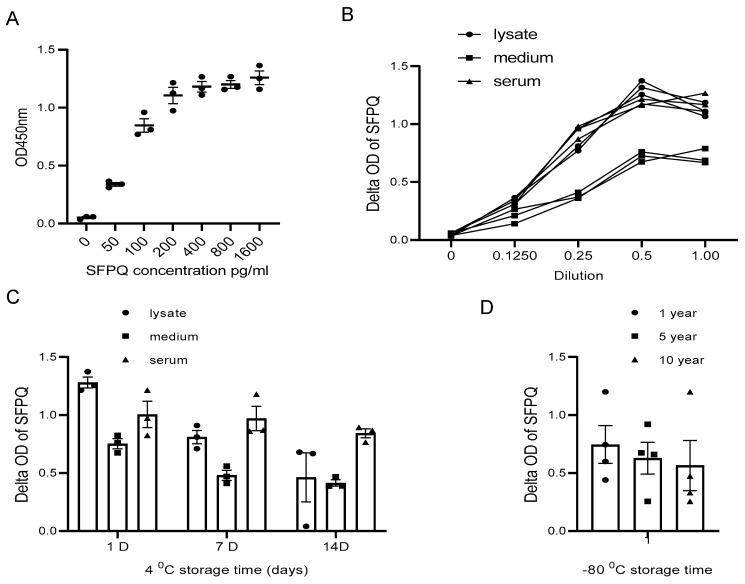
SFPQ BA-ELISA is highly sensitive and reliable. ELISA can detect SFPQ in samples of 20 pg or greater (**A**). ELISA curves obtained with serial dilution of SFPQ recombinant samples. ELISA with 2F8- or 2D5-coated mAb was undertaken for serial dilution of SFPQ protein (on the X-axis, 1.0 corresponds to undiluted sample). OD was then read at 450 nm. This ELISA experiment was conducted several times for recombinant SFPQ and native SFPQ (standard curve). One representative experiment is shown here. (**B**). Detection of SFPQ in cancer cell lysates, conditional media, and human sera of patients with cancer in serial dilution. NSCLC A549, H661, and H846 cells analyzed by ELISA with coated 2F8 mAb. Cell lysate, cell culture medium, and 3 NSCLC sera samples were tested. (**C**). Detection of SFPQ in samples with different storage times at 4 °C. Sample storage time is indicated. (**D**). Detection of SFPQ in samples with different storage times at −80 °C. Serum samples were selected randomly from samples with different storage durations. Each of the experiments shown in this figure was conducted at least twice.

The assay sensitivity reached a low pg/mL level, so the diluted cell lysate and cell culture medium samples could suit the assay better for SFPQ measurement (Figure 5A,B).

The sample type is another factor that needs to be considered in our assay application.

SFPQ was not stable in the cell lysate and the cell culture conditioned medium for long-term storage at 4 °C (Figure 5C). With the Bionet samples collected between 2009 and 2023, the sample set at different storage times demonstrated that the SFPQ levels in the samples stored in a bank for approximately 10 years at −80 °C are comparable to those measured in the fresh samples (Figure 5D). These results showed no significant changes in the SFPQ levels after sample collection and storage freezing at −80 °C.

### 2.4. Serum Collection from Patients with Cancer and Other Donors

The serum samples used in this study were obtained from patients with lung cancer or other solid cancers who were prospectively recruited between January 2009 and January 2022. This study was approved by the Institutional Ethics Committee Review Board (IRB# 9709 M00126) of the University of Minnesota. Some samples were collected by Bionet of the University of Minnesota Medical Center, and the rest of the samples were purchased from Accio Biobank USA. The blood was sampled before or during the surgical diagnostic or therapeutic procedures, and the serum was recovered and stored at −80 °C.

### 2.5. Patient Characteristics

The serum samples were collected from 132 patients with NSCLC (*n* = 46, lung cancer *n* = 52), breast cancer (*n* = 18), prostate cancer (*n* = 12), thyroid cancer (*n* = 7), kidney cancer (*n* = 15), or hepatic cancer (*n* = 9), or other non-cancer diseases. The patient population with NSCLC consisted of 15 men and 21 women who had a median age of 67.5 years (range, 38–91 years) and a mean age of 68.9 years (standard deviation, 13.5). The histological subtypes of NSCLC were divided into the differentiated (*n* = 17) and undifferentiated (*n* = 29) subtypes. According to the eighth edition of the *Union for International Cancer Control* (UICC) classification, 13, 16, and 17 patients with NSCLC had clinical stages II, III, and IV, respectively.

**Figure 6:**
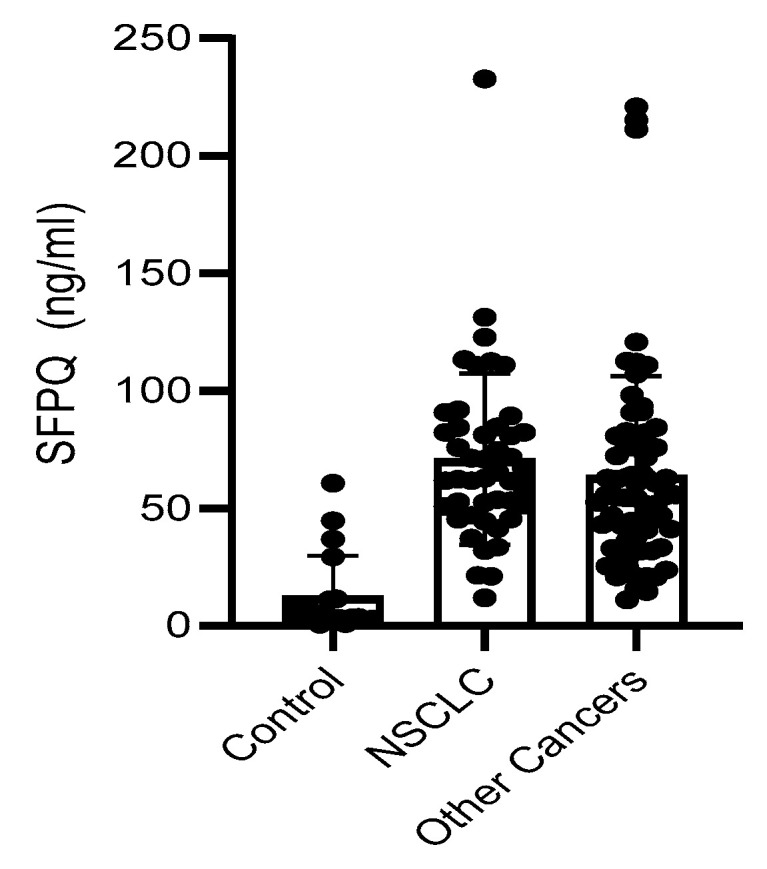
SFPQ BA-ELISA is reliable. Detection of SFPQ in human serum of patients with NSCLC or other solid cancers. ELISA was with coated 2F8/2D5 mAb. Serum samples were collected and stored at −80 °C from multiple sites with different storage durations. Each of the experiments shown in this figure was conducted at least twice.

SFPQ level is high in cancer serum. We employed a set of serum samples for the assay we developed. We measured the SFPQ levels in the serum samples via ELISA. The samples were diluted 10-fold, and then subjected to SFPQ ELISA. SFPQ was shown to be more abundant in the patients with cancer than in the controls (Figure 6). The SFPQ level detected by ELISA in the control group (12.47 ± 17.24 ng/mL) was much lower than that in the lung cancer (70.97 ± 35.24 ng/mL) and other cancer serum groups (66.57 ± 41.64 ng/mL, respectively). The serum SFPQ levels in the other cancer groups were varied among them, but the case numbers were small (Table 1). Together, these data indicate that the SFPQ concentration is greater in the serum of patients with lung cancer and patients with other cancers than in the serum of patients with other diseases. Regarding NSCLC, when we set the 40 ng/mL as the cut off, the serum SFPQ levels distinguished the healthy controls from the patients with NSCLC with an area under the curve (AUC) of 0.876, a sensitivity of 87%, and a specificity of 94%. For the other cancers tested, the serum SFPQ level distinguished the healthy controls from the patients with cancer with an AUC of 0.803, a sensitivity of 85%, and a specificity of 95%.

## 3. Discussion

Cancer markers are measurable molecules that circulate in the blood or tissue and are generated by tumor cells or immune cells in response to cancer progression [3,4,21]. They play an important role in clinical diagnosis, prognosis, and therapy monitoring. Although DNA, RNA, and even physical images have been used, proteins remain the most common marker type. Serum cancer markers have several advantages over the traditional diagnostic approaches, including being less expensive, less time-consuming, and less invasive. NSCLC is one of the most common cancers. Numerous clinical trials have been conducted to identify the markers for advanced-stage NSCLC. Some markers, including CEA, CA19-9, CA125, AFP, NSE, CK20, CDX2, STAT3, CA15-3, and CYFRA21-1, have been investigated, but none were found to be specific for cancer [2,22,23]. SFPQ is a multifunctional protein. It has the ability to bind to DNA and RNA, and thus regulate RNA splicing and protein transcription [10,11,12,24]. It was also discovered that its accumulation in the cytoplasm could be the cause of some neuronal diseases [12,16]. The SFPQ levels are elevated not only in lung cancer, but also in other cancer samples. The SFPQ levels are greater in cancer cells than in other cells. Immunocytochemistry revealed similar results. We used an ELISA to detect SFPQ in cell lysates and cell conditioned media. The SFPQ concentration was high in the cancer cell medium, suggesting that SFPQ is released from the cells. The high growth rate and high turnover rate of cancer cells could be the reason for the high SFPQ level in the body fluid of the patients with cancer, which further suggests that SFPQ is a diagnostic marker.

Specific SFPQ antibodies were developed and used for the ELISA. The assay was stable and sensitive for SFPQ measurement. The samples from different storage conditions and time periods were tested, and the results demonstrated that the assay is stable and potentially suitable for further clinical use. The serum SFPQ results revealed high specificity for NSCLC and the other solid cancers. The next step for this study will be the observation of dynamic changes in the SFPQ level with the cancer progression stages. Although these data may differ among large-scale samples, SFPQ is a valuable diagnostic marker.

Our findings suggest that SFPQ may serve as a diagnostic marker for lung cancer and other solid cancers. The sandwich ELISA described here is suitable for further clinical application. More research will be required to determine whether this will hold true with a larger number of patient cases and samples.

## 4. Materials and Methods

### 4.1. Monoclonal Antibodies Specific for SFPQ

Mouse immunization and hybridoma generation were conducted by following the previously published methods. The antigen used was purified native human SFPQ. It is also used to screen SFPQ antibody-producing hybridomas. The hybridomas that were found to be positive upon screening on SFPQ-coated plates were further tested for recombinant SFPQ (Abnova, Taipei, Taiwan). Then, the isotype of eight positive clones was determined using an mAb-based Ig isotyping kit (BD Pharmingen). Among them, only 8 (1A6, 2A4, 2C1, 2D5, 2F8, 3A7, 3D5, and 4A1) recognized both recombinant human SFPQ and native SFPQ purified from human cells. 2C1, 2D5, and 3A7 were shown to be the only mAbs able to detect by IHC.

### 4.2. mAb Biotinylation

The antibodies were biotinylated using the EZ-Link^®®^ Micro NHS-PEG4-Biotinylation Kit (Thermo Scientific, Waltham, MA, USA) by following the manufacturer’s instructions. The purified SFPQ IgG was labeled with biotin at a 1:6 ratio. Biotinylation was quantified with the Biotin quantification kit (Promega, Madison, WI, USA). A total of 100 µg of SFPQ IgG was labeled with biotin at an average of 4.23 nM (IgG1: 4.26/1A6, 4.19/3A7, 4.25/2C1, 4.31/2D5; IgG2: 4.22/3D5, 4.16/2F8; IgG3: 3.56/2A4). Then the labeled antibodies were used for assay development.

### 4.3. SFPQ and SFPQ Antibody Purification

SFPQ was purified with an SFPQ affinity column. Sepharose 4B beads (17090601, GE Healthcare) and antibodies (P23246, Raybiotech, Peachtree Corners, GA, USA; HPA054689, Sigma, USA and 67129-1-Ig, Proteintech, Rosemont, IL, USA) were used to make affinity columns. A total of 500 ug of each above antibody was coupled with beads by following the manufacturer’s instruction, and the beads were then used to pack an affinity column for SFPQ purification. Briefly, the collected medium was centrifuged (4000× *g* for 5 min at 4 °C) and filtered for clarification (through a 0.45 μm filter), and the protein was purified by affinity chromatography using chelating Sepharose (GE Healthcare) equilibrated in 20 mM Na phosphate (pH 7.4) and 500 mM NaCl. SFPQ was eluted with 2M Glycine (pH2.8) and neutralized with Tris-HCl (2M, pH 8.2). Fractions containing SFPQ were determined by Western blotting using 2F8 mAb, pooled, concentrated, and buffer-exchanged into 20 mM HEPES (pH 7.2) and 150 mM NaCl using a Millipore Ultrafree-15 spin concentrator. The protein concentration was monitored by spectrometry (280 nm absorbance). This procedure led to the purified native SFPQ protein, which we used development antibody. Antibody purification using a similar protocol with Protein G beads was performed.

### 4.4. Development of the Sandwich ELISA Test to Detect SFPQ

For conventional ELISA (Figure 3), the SFPQ antibody was coated with HRP-anti-mouse or rabbit conjugate (2 μg/mL, 15585-1, Proteintech, Rosemont, IL, USA) for detection (3 μg/mL H00006421, Abnova). For biotin–avidin (BA)-ELISA, the first step involved coating 96-well microtiter plates (NuncSorb, Thermo Scientific, Waltham, MA, USA) with mAbs (100 μL at 5 μg/mL in PBS, 4 °C ON). After three washes with PBS, the wells were saturated with BSA (200 μL, 3% in PBS, at least 2 h at RT). After three washes with PBS, 100 μL of the sample containing SFPQ protein was added to the wells, and the protein was allowed to bind to its specific mAb for 3 h at RT. All the proteins not specifically bound were eliminated by four washes with PBS. Then, the biotinylated mAb was added (100 μL at 1 μg/mL in PBS, 45 min at RT). After three washes with PBS, streptavidin–HRP (Beckman) was added (100 μL, 1/1000 in PBS, 1% BSA, 30 min at RT). After three washes with PBS, 100 μL of HRP substrate (TMB: 3,3′,5,5′-tetramethylbenzidine; Thermo Scientific Pierce) was added, and the reaction was allowed to progress for 15 min in the dark. The reaction was then stopped by the addition of 100 μL of 2 M H_2_SO_4_. The results were obtained at 450 nm with a plate reader.

### 4.5. Cell Culture

Cell lines from the ATCC were used in this study. RWPE-1 and HBCE3-KT cells were cultured with Keratinocyte-SFM (Gibco 10724-011) and 0.1% soybean trypsin inhibitor according to the manufacturer’s instructions. PCS-201-020, HEK293, MHCC97, Caki-1, MDA231, and DU145 cells were cultured in DMEM supplemented with 5% FBA. Primary lung cell lines were established from patients who fulfilled the diagnostic criteria for lung cancer and other lung diseases, including the pathological diagnosis of usual interstitial pneumonia. The patient controls were selected to be similar in age to the patients with non-cancer lung disorders. Control and lung cancer cell lines were derived from lungs and cultivated as previously described [25]. Human patient tissue sections were collected and prepared by Bionet, the University of Minnesota. We utilized 4 non-cancer primary control cell lines from lung tissues not involved in the primary process: histologically normal lung tissue from a gunshot victim (*n* = 1) or chronic obstructive pulmonary disease (COPD) (*n* = 3) and 12 tissue samples from patients without cancer with interstitial lung disease (*n* = 5) or chronic obstructive pulmonary disease (COPD) (*n* = 7) for control tissue sections. A pathologist verified that all the tissues were tumor-free.

### 4.6. Serum Collection from Patients with Cancer and Other Donors

The serum samples used in this study were obtained from patients with lung cancer or other solid cancers who were prospectively recruited between January 2009 and January 2023. This study was approved by the Institutional Ethics Committee Review Board (IRB# 9709 M00126) of the University of Minnesota. Some samples were collected by Bionet of the University of Minnesota Medical Center, and the rest of the samples were purchased from Accio Biobank, Newmarket, UK. 

Western blot analysis. Cells were washed twice in cold PBS and lysed in New RIPA lysis buffer (150 mM NaCl, 50 mMTris pH 8.0, 1 mM EDTA, 1 mM EGTA, 0.5% sodium deoxycholate, 0.1% SDS, and 1% Triton X-100) supplemented with a protease inhibitor cocktail (0.1 M phenylmethylsulfonyl fluoride, 5 μg/mL leupeptin, 2 μg/mL aprotinin, and 1 μg/mL pepstatin). The protein concentrations of the whole-cell lysates were determined using the BCA method, and equal amounts of each protein sample (15 μg) were separated on 8~14% SDS–polyacrylamide gel at 80 V. The separated proteins were then transferred to a polyvinylidene difluoride membrane for 8 min on a Turbo transfer system (Invitrogen, Waltham, MA, USA). After blocking with 5% skim milk powder for 1 h at RT, the membrane was incubated with primary antibody for 1 h at RT or overnight at 4 °C. The membrane was washed three times for 15 min with 0.05% PBS-Tween, and then incubated for 1 h at RT with a horseradish peroxidase-conjugated secondary antibody. After extensive washing with 0.05% PBS-T, the protein bands were visualized by ECL Plus according to the manufacturer’s instructions (Cell Signaling Technology, Beverly, MA, USA).

Real-time reverse transcription PCR. Total RNA was extracted with the RNeasy Mini Kit, and cDNA was synthesized with the miScript92 RT Kit (Qiagen, Hilden, Germany). The PCRs contained 10 μL of SYBR Green SuperMix (Bio-Rad, Hercules, CA, USA), 900 nM forward primer, 900 nM reverse primer, and 50 ng of cDNA in a 20 μL reaction volume. GAPDH was used as a reference, and the expression of GAPDH was normalized to 1. Reactions were performed in an a7900 HT Sequence Detector (Applied Biosystems, Waltham, MA, USA) with the cycling protocol described previously (Applied Biosystems, Waltham, MA, USA). The primers used were as follows:

GAPDH Forward: 5′- TGTTGCCATCAATGACCCCTT-3′

GAPDH Reverse: 5′-CTCCACGACGTACTCAGCG-3′

SFPQ Forward: 5′-GATCTACAGGGAAAGGCATTGTTG-3′

SFPQ Reverse: 5′-GATACATTGGATTCTTCTGGGCA-3′

RT–PCR products were quantified in the log-linear portion of the curve using LightCycler analysis software (Roche, Basel, Switzerland, https://diagnostics.roche.com/global/en/products/instruments/lightcycler-480-ins-445.html, accessed on 1 July 2024) and compared to an external calibration standard curve.

Statistical analysis. All the experiments were performed at least in triplicate, and the results were analyzed using Student’s t-test or two-way ANOVA (for the proteomics methods described above). The criterion for significance was *p* < 0.05. Numerical data are reported as the means ± standard deviations.

## Figures and Tables

**Table 1:** SFPQ level in human serum measured by BA-ELISA.

Disease	Case Number	Gender	Cancer Stage	SFPQ (ng/mL)	Stdev
Non-Cancer	19	M:12; F7		12.47	17.3
NSCLC (Lung cancer)	46(52)	M:32(4),F:14(2)	II:13,III:16,IV:17	70.96	36.25
Kidney Cancer	15	M:8, F:7	II:2,III:8,IV:5	66.84	47.03
Prostate Cancer	12	M:15	II:3,III:6,IV:3	53.27	44.9
Liver Cancer	9	M6,F3	II:1,III:4,IV:4	80.16	41.44
Breast Cancer	18	M:1, F17	II:3,III:11,IV:4	50.27	44.3
Thyroid Cancer	7	M:2,F5	II:1,III:1,IV:2,V:2,VI:1	61.35	48.4

SFPQ value obtained with BA-ELISA is described in the Materials and Methods Section. Purified native SFPQ was used for standard curve creation.

## Data Availability

The data that support the findings of this study are available on request from the corresponding author.
